# Schistosomiasis Vector Snails and Their Microbiota Display a Phylosymbiosis Pattern

**DOI:** 10.3389/fmicb.2019.03092

**Published:** 2020-01-31

**Authors:** Camille Huot, Camille Clerissi, Benjamin Gourbal, Richard Galinier, David Duval, Eve Toulza

**Affiliations:** IHPE, Univ. Montpellier, CNRS, Ifremer, Univ. Perpignan Via Domitia, Perpignan, France

**Keywords:** microbiota, phylosymbiosis, metabarcoding, Planorbid snails, tripartite interactions, schistosomiasis

## Abstract

Planorbidae snails are the intermediate host for the trematode parasite of the *Schistosoma* genus, which is responsible for schistosomiasis, a disease that affects both humans and cattle. The microbiota for *Schistosoma* has already been described as having an effect on host/parasite interactions, specifically through immunological interactions. Here, we sought to characterize the microbiota composition of seven Planorbidae species and strains. Individual snail microbiota was determined using 16S ribosomal DNA amplicon sequencing. The bacterial composition was highly specific to the host strain with limited interindividual variation. In addition, it displayed complete congruence with host phylogeny, revealing a phylosymbiosis pattern. These results were confirmed in a common garden, suggesting that the host highly constrains microbial composition. This study presents the first comparison of bacterial communities between several intermediate snail hosts of *Schistosoma* parasites, paving the way for further studies on the understanding of this tripartite interaction.

## Introduction

A microbiota consists of microbial communities in association with a host. Here, we defined the microbiota as all microorganisms involved in a long-lasting interaction with a host, excluding parasites and pathogen microorganisms (Bordenstein and Theis, 2015). The microbiota is involved in numerous functions, including nutrition (McCutcheon et al., 2009), development (McFall-Ngai, 2002; Fraune and Bosch, 2010), reproduction (Perlman et al., 2008; Werren et al., 2008), and immunity (Lee and Mazmanian, 2010; Hahn and Dheilly, 2016). For example, the bacterial microbiota of the mosquito gut is involved in the immune response of its host against dengue pathogen virus, through direct inhibition of the virus toward bacterial metabolites as well as through indirect effects by stimulating its basal immunity (Saraiva et al., 2016). This illustrates the importance of considering microbiota in host–pathogen interactions.

Numerous studies have already explored the factors shaping microbiota composition in several models and highlighted the role of neutral processes (Burns et al., 2015), environment (Roder et al., 2015), host genetic background (Brucker and Bordenstein, 2011), or host physiology/immunity (Chu and Mazmanian, 2013; Hahn and Dheilly, 2016). Results from these studies demonstrate the effect of the host immune system in microbiota homeostasis. In *Hydra*, the nature and combination of antimicrobial peptides belonging to the arminin family are involved in the species specificity of host microbial communities that follow host phylogeny (Franzenburg et al., 2013).

Here, we characterized the microbiota of several genera of Planorbidae, a family of freshwater snails. These snails are the intermediate hosts for the parasite *Schistosoma* spp., a genus of trematode parasites which develop asexually in the snails before infecting vertebrates where sexual reproduction takes place. Human *Schistosoma* species, mainly *Schistosoma mansoni*, *Schistosoma haematobium*, and *Schistosoma japonicum*, infect about 250 million people (Hotez et al., 2014) annually, and each year, more than 200,000 people die as a result of the infection worldwide (WHO, 2019). While *Biomphalaria glabrata* and *Biomphalaria pfeifferi* snails can be infected with *S. mansoni* (responsible for human intestinal schistosomiasis), *Planorbarius metidjensis* is responsible for the transmission of *Schistosoma bovis*, and *Bulinus truncatus* snails are natural hosts for *S. bovis* as well as *S. haematobium* (agent of the human urinary schistosomiasis). Interestingly, it has been shown that within the *B. glabrata* species, some strains can be completely refractory to infection depending on the parasite strain, a phenomenon called compatibility polymorphism (Theron et al., 2014; Galinier et al., 2017). These snail–parasite interactions, resulting from coevolution dynamics, reflect differences in host immune capacities or differences in immunobiological interactions between different host–parasite combinations.

The snail immune response in this interaction is complex with a specificity according to the parasite strain (Portela et al., 2013). Indeed, snails’ immune effectors and receptors seem to be specific to the parasite, and the type (cellular or humoral) and efficiency of immune response is linked to the infection type [primo-infection or challenge (homologous or heterologous)] (Pinaud et al., 2016). A shift in microbiota composition following an infection was observed after an immune challenge, where humoral immunity took place (Portet et al., 2018). This highlights the importance of further in-depth studies of the relationship between the host’s immune and vectorial capacities and its microbiota composition. To do this, it is essential to first characterize the factors that shape microbial communities and their host specificity.

To identify the effect of host identity in bacterial microbiota composition, we used 16S ribosomal DNA amplicon sequencing to analyze the bacterial communities at the individual level (10–15 samples per condition/strain) for seven snail strains: four different strains of *B. glabrata*, one strain of *B. pfeifferi*, one strain of *P. metidjensis*, and one strain of *B. truncatus*. Our results provided the first characterization of microbiota for several strains of mollusks, the intermediate hosts of the *Schistosoma* sp. parasite.

## Materials and Methods

### Rearing Conditions

#### Individual Tank Experiment

To determine the bacterial microbiota composition and specificity, we used four strains of *B. glabrata*, one from Guadeloupe (B. gla GUA2), two from Brazil (B. gla BAR2 and B. gla BRE2), and one experimentally selected for reduced compatibility to different *S. mansoni* parasite strains (B. gla BS902) (Theron et al., 2014). In addition, *B. pfeifferi* (Oman) as well as another Planorbinae genus, *P. metidjensis* (Spain), and a Planorbidae non-Planorbinae species, *B. truncatus* (Spain), were used (Table 1).

**TABLE 1:** Origin of snail strains used in this study.

**Species**	**Strain**	**Strain code**	**Strain origin**
*Biomphalaria glabrata*	BAR2	B. gla BAR2	Belo Horizonte, Brazil (Oliveira, 2013)
*Biomphalaria glabrata*	BRE2	B. gla BRE2	Recife, Brazil (Théron, 1975)
*Biomphalaria glabrata*	GUA2	B. gla GUA2	Dans Fond and Guadeloupe (2005)
*Biomphalaria glabrata*	BS902	B. gla BS902	Salvador, Brazil (1960)
*Biomphalaria pfeifferi*	/	B. pfe	Anakhar, Oman (Moné and Moné, 2015)
*Planorbarius metidjensis*	/	P. met	Salamanca, Spain (Mas-Comà, 2014)
*Bulinus truncatus*	/	B. tru	Almeria, Spain (Olega, 2015)

All strains were reared in the same conditions: 20 individuals of each strain were maintained in separate tanks (3 L) and fasted 1 week before sampling to avoid changes in microbiota composition associated with diet. Snail shell size (diameter, 7–8 mm), which is directly correlated to age, was similar for each experimental group.

#### Common Garden Experiment

Thirty mollusks of each strain were maintained for 2 months within the same 8-L tank, where perforated baskets separating the strains were used to avoid mixing and potential antagonistic interactions, but which favored the potential exchange of microbiota as they were reared in the same tank. Mollusks were fed with lettuce every 2 days (and fasted 1 week before sampling), and 50% of the water was renewed weekly.

### Sampling

The mollusk shells were cleaned with cotton buds soaked in bleach (to avoid transfer of contaminants), and mollusks were then removed from the shell by dissection and flash frozen individually in liquid nitrogen before being kept at −80°C until DNA extraction.

### DNA Extraction and Sequencing

DNA was extracted with the Nucleospin^®^ tissue extraction kit from Macherey-Nagel and quantified with a Qubit 2.0 Fluorometer following the procedure described in the Qubit^TM^ dsDNA HS Assay Kit, to check its purity and yield.

For samples with highest DNA yield and quality (11–15 depending on snail strains for individual tank experiment, and 10 per strain for the common garden experiment, Supplementary Table S1), 16S ribosomal (rRNA) gene (V3–V4 regions) (Klindworth et al., 2013) libraries were generated using PCR primers 341F (5′-CCTACGGGNGGCWGCAG-3′) and 805R (3′-GACTACHVGGGTATCTAATCC-5′) following the standard Illumina two-step procedure. Libraries were paired-end sequenced with 250-bp read length on three different flow cells using the MiSeq system (Illumina) at the Génome Québec Innovation Centre, McGill University, Montréal, Canada. A blank sample was sequenced in each of the three runs, but as very few sequences were obtained, this dataset was not further analyzed.

### Analysis of 16S Sequences

The Find Rapidly OTU with Galaxy Solution pipeline implemented on a galaxy instance^1^ was used for data processing (Escudié et al., 2017). In brief, paired reads were merged using FLASH (Magoč and Salzberg, 2011). After denoising and primers/adapters removal with CUTADAPT (Martin, 2011), *de novo* clustering was performed using SWARM with a local clustering threshold (Mahé et al., 2014), with aggregation distance *d* = 3 after denoising. Chimeras were removed using VSEARCH (Rognes et al., 2016). We filtered the dataset for singletons and performed affiliation using Blast + against the Silva database (release 128, September 2016) for 16S rRNA gene amplicons. Finally, operational taxonomic unit (out) tables were produced in a standard BIOM format for subsequent analyses.

We then used the packages phyloseq 1.24.2 (Mcmurdie and Holmes, 2013) and vegan 2.5-4 (Oksanen et al., 2019) with RStudio (R Core Team, 2017). Sample B. gla BRE _JC_7 had too low coverage (155 reads) and was thus discarded from subsequent analyses. Non-bacterial sequences as well as singletons remaining after all the secondary filtering steps were discarded from the dataset. We rarefied the data according to the sample with fewer sequence numbers (18,299 reads for the Individual Experiment and 15,969 reads for the Common Garden) to normalize for sequencing coverage. We characterized the beta-diversity dissimilarities using principal coordinates analyses (PCoA) and hierarchical clustering on Bray–Curtis (BC) distance matrix (ranging from 0 for identical communities to 1 for completely different communities).

### Core Microbiota

To determine the core microbiota, which is the most stable part of the microbiota, we identified the families and genera that were either present in 100% of individuals or absent from a maximum of one individual for each strain, and represented at least 0.5% of sequences for each strain.

### Snail Phylogeny

Phylogenetic analysis was performed using 28S rRNA gene sequences from the National Center for Biotechnology Information database (*B. glabrata*, AF435694.1; *B. pfeifferi*, MG461588.1; *P. metidjensis*, AF435671.1; and *B. truncatus*, AF435659.1). The 28S rRNA gene sequence of a Physidae species, *Physa* sp. (Egypt) (sister family of the Planorbidae) was used as an outgroup (AF435654.1). The sequences were aligned using MUSCLE (v3.8.31), and the tree was reconstructed using the maximum likelihood method implemented in the PhyML program (v3.1/3.0 aLRT) with 500 bootstraps on^2^ (Dereeper et al., 2008, 2010).

### Statistical Analyses

We analyzed the variance due to host effect on dissimilarity matrices using permutational multivariate analysis of variance. Permutational multivariate analyses of variance were done with 999 permutations. For all analyses, the threshold significance level was set at 0.05.

We used an indicator value index and 999 permutations (multipatt, {indicspecies}) (Cáceres and Legendre, 2009) to identify OTUs associated with the different host species.

*P* values were corrected for multiple comparisons using Benjamini and Hochberg’s method (Benjamini and Hochberg, 1995) (p.adjust, {stats}).

## Results

### Bacterial Composition

**FIGURE 1:**
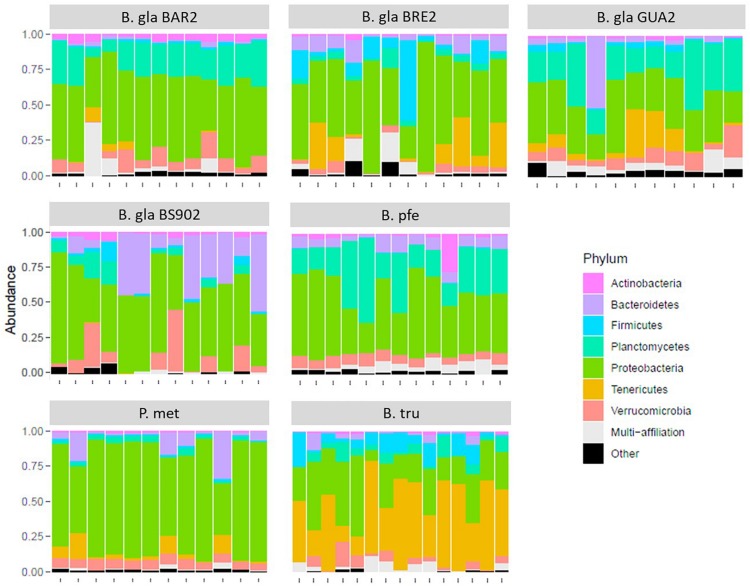
Relative composition for the seven most abundant phyla in the microbiota for each strain of mollusk.

At the phylum level, the composition of bacteria was similar in all *Biomphalaria* samples (Figure 1), with Proteobacteria being the predominant phylum for the different strains, in which the Flavobacteriaceae, Rhodobacteraceae, Comamonadaceae, and Xanthomonadacea families were the most represented. This is consistent with the results found by Portet et al. (2018). *P. metidjensis* composition also displayed a high proportion of Proteobacteria, in particular Alphaproteobacteria, represented by Rhodobacteraceae (Supplementary Table S1). In the case of *B. truncatus*, more pronounced differences were visible at the phylum level, where Proteobacteria and Tenericutes were dominant (Figure 1), with the latter represented mainly by Mycoplasmataceae, and more specifically by the genus *Mycoplasma* (Supplementary Table S1).

The core microbiota was determined as all bacterial families that were either present in 100% of individuals or absent from a maximum of one individual. The core microbiota was composed of 44 families, for all strains included (Supplementary Table S2). The core microbiota composition varied between strains, whereas seven bacterial families were common to all strains, where *Cloacibacterium* (a Flavobacteriaceae genus) was found as part of the core microbiota in all *Biomphalaria* strains and species, except for *B. glabrata* BS90, which was absent in the core microbiota of *P. metidjensis* and *B. truncatus*.

### Beta-Diversity, Ordination, and Clustering

**FIGURE 2:**
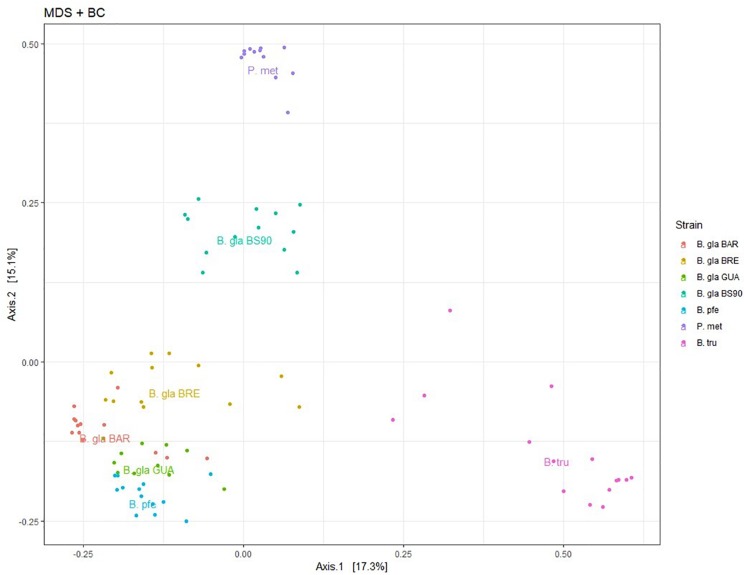
Principal coordinate analysis (PCoA) on Bray–Curtis dissimilarity matrix for bacterial microbiota composition. Each dot is an individual and each color, a strain. The labels are displayed at the barycenter.

An ordination using PCoA was performed on BC distance matrix to visualize the similarities between individuals according to their bacterial composition (Figure 2). The first two axes explained 32.4% of the variability observed. Individuals tended to group according to host species. *Biomphalaria* strains were grouped at the exception of the *B. glabrata* BS902 strain. Individuals of the two other species, *P. metidjensis* and *B. truncatus* were separated from *Biomphalaria* individuals.

**FIGURE 3:**
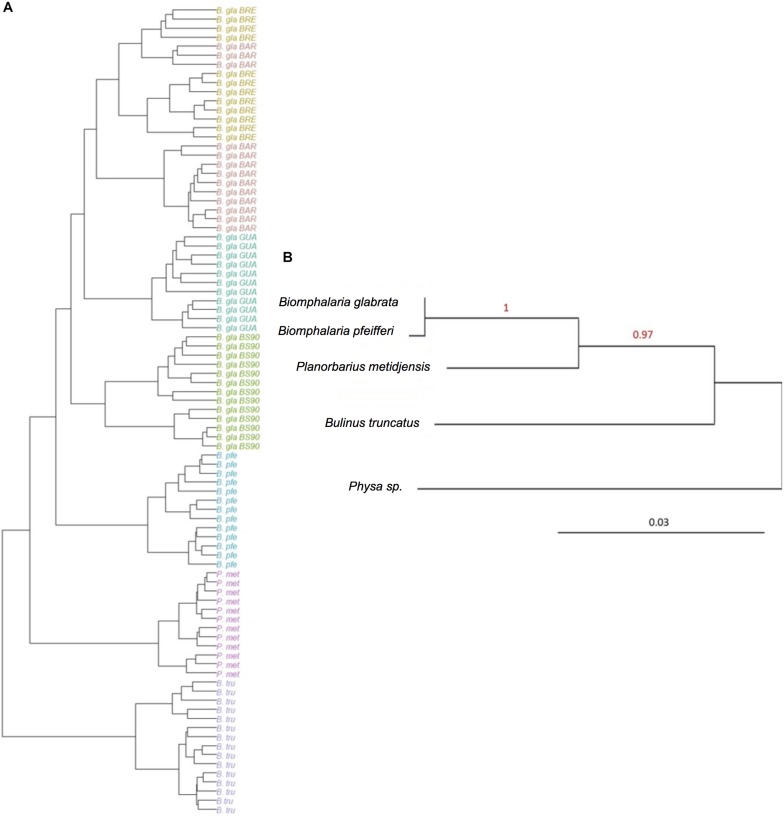
**(A)** Hierarchical clustering based on Bray–Curtis dissimilarity matrix and Ward linkage for OTUs of the core microbiota. Each color represents a strain. **(B)** Phylogenetic tree of host species based on 28S rRNA gene sequence and using maximum likelihood with 500 bootstraps (%) for node support. *Physa* sp. was used as an outgroup. The red numbers are the bootstrap values for the nodes.

The hierarchical clustering analysis based on BC distance on the core microbiota confirmed a grouping between individuals of the same strain or species. Moreover, the dendrogram of bacterial communities reflected host phylogeny (Figure 3). *B. truncatus* and *P. metidjensis* were separated from *Biomphalaria* species, and *B. pfeifferi* was separated from *B. glabrata* strains. The microbiota specificity according to host genetic background was confirmed by MANOVA on BC dissimilarity matrix on core microbiota (*P* < 0.001).

The core microbiota beta-diversity was analyzed using the same approach with a PCoA ordination based on the BC dissimilarity index (Figure 3). Individuals belonging to the same strain tend to cluster together, and *Biomphalaria* strains were grouped, except for, again, the BS902 strain. In addition, individuals from the two other species, *P. metidjensis* and *B. truncatus*, were separated from *Biomphalaria* individuals. This analysis of the core microbiota composition confirmed the pattern obtained for the whole microbiota with specific core microbiota associations for individuals belonging to the same phylogenetic group (strain, species, or genus), suggesting a phylosymbiosis pattern, driven by host species among snail intermediate hosts of schistosomiasis.

We used indicator value index and permutation tests to identify OTUs significantly associated with each host species. On average, each species had 37 specific OTUs belonging to 88 genera (Supplementary Table S3). Although the 88 genera were mostly (77%) specific of each host species, this analysis highlighted that specific OTUs belonging to five genera (*Pirellula*, *Planctomyces*, *Candidatus Odyssella*, *Mesorhizobium*, and *Pseudomonas*) were found in more than 50% of host species. Strikingly, specific OTUs from *Mesorhizobium* and *Pseudomonas* showed identical distribution within host species (presence in BRE, BS90, Pfe, and Plan), suggesting that these bacteria might cooperate within host microbiome.

### Common Garden

Environmental conditions and/or host genetics can both affect microbiota composition. To investigate the main key drivers for core microbiota composition, we performed an additional experiment with all strains raised together in the same water for 2 months. However, we could not include *B. pfeifferi* in this analysis because most individuals did not survive until the end of the experiment, as they escaped their basket and were predated.

The PCoA ordination revealed a similar microbiota specificity by strain to those observed in the first experiment with a grouping by strain then species (Supplementary Figure S1), as confirmed by MANOVA analysis on host effect on BC dissimilarities between host strains (*P* < 0.001).

## Discussion

To understand the host effect in shaping microbiota in Planorbidae schistosomiasis vector snails, we characterized the individual bacterial communities associated with several strains of *B. glabrata*, *B. pfeifferi*, *P. metidjensis*, and *B. truncatus* snails. Working on individuals reared in lab conditions favored the control of most of the parameters that can influence microbiota composition.

In the present study, the whole microbiota was characterized using 16S amplicon sequencing. We identified 31,207 OTUs among the seven different snail strains. Most of OTUs were not assigned to the species level, and 63% were assigned to the genus level. This corresponds to the limitation of the 16S V3V4 marker resolution. In addition, the Blast + -based pipeline we use for taxonomic affiliation avoids false affiliation when a sequence matches with several sequences in the database. If several Blast results have identical scores for a given OTU, and these taxonomies differ across hits, the OTU is set to “multi affiliation” (Escudié et al., 2017).

A few studies have characterized the cultivable flora of *B. glabrata* and have identified Aeromonadaceae, Enterobacteriaceae, Moraxellaceae, and Pseudomonadaceae as being the most prevalent bacterial families in this species (Ducklow et al., 1979, 1981; Silva et al., 2013). The dominant families described in the previous studies were also represented in our dataset. However, the relative composition of microbiota at the phylum level revealed that Proteobacteria were dominant for most of the different *Biomphalaria* strains, represented by three main families: Rhodobacteraceae (Alphaproteobacteria), Comamonadaceae (Betaproteobacteria), and Xanthomonadaceae (Gammaproteobacteria). Not all bacterial families can easily be cultivated; the MiSeq technology allows identifying the whole bacterial diversity. Our results are consistent with those found for *B. glabrata* BRE bacterial microbiota in Portet et al. (2018), in which these three families were the most abundant of the core microbiota. The microbiota of a Guadeloupian strain of *B. glabrata* was also described using a similar approach (Allan et al., 2018), and similarly, the dominant phyla were Proteobacteria and Bacteroidetes.

Proteobacteria have been described as key factor in marine bivalve digestion, like the great scallop *Pecten maximus*, as they are involved in the degradation of major alimentary components contained in their diet (Lasa et al., 2016). This phylum is also dominant in other mollusks, as is the case for oysters *Crassostrea corteziensis*, *Crassostrea gigas*, and *Crassostrea sikamea* (Trabal et al., 2012). As this is the first study to characterize the bacterial microbiota of *B. glabrata* BS902, *P. metidjensis*, *B. truncatus*, it is not possible to compare with previous results and to draw any definitive conclusions.

Interestingly, the bacterial families that comprise the core microbiota for each mollusk strain were also among the most abundant taxa in the whole microbiota, which is consistent with previous studies on this model (Portet et al., 2018). In corals, for example, the OTUs belonging to core microbiota are among the rare taxa and are difficult to detect within the whole microbiota (Ainsworth et al., 2015). Owing to the high interindividual variation, in some studies, the core microbiota in the coral model was defined by OTUs present in a limited proportion of individuals, 30% in Ainsworth et al. (2015) and 50% in Brener-Raffalli et al. (2018). In our study, the most impressive case of high abundance in core taxa concerns *B. truncatus*, with the Mycoplasmataceae family, and more precisely the genus *Mycoplasma*, which represents more than 47% of the whole microbiota. The genus *Mycoplasma* was originally described as an obligate vertebrate parasite and the causative agent of human genital and respiratory diseases with a high tissue specificity (Razin et al., 1998). This genus has been described in other models including algae and several invertebrates such as oysters (King et al., 2012; Clerissi et al., 2018), abalone (Huang et al., 2010), and sacoglossans (Davis et al., 2013). It has also been described as being one of the most abundant microorganisms in the deep-sea bone-eating snail, *Rubyspira osteovora* (Aronson et al., 2016). Its role in these organisms remains unclear, but some authors hypothesized that they may help with digestion (Fraune and Zimmer, 2008; Duperron et al., 2012; Aronson et al., 2016), notably because of its presence in the digestive tract. In the present study, bacteria belonging to the genus *Cloacibacterium* have been found in all *Biomphalaria* strains and species, and *C. haliotis* has been described in another mollusk, the sea snail *Haliotis discus* (Hyun et al., 2014).

The results of dissimilarity between strains revealed that the bacterial microbiota of *B. truncatus* individuals was distinct from other species with most of the BC distance values ranging between 0.8 and 0.99. The phylogenetic distance of this species from the others could explain this difference. Indeed, this is the only species, in this study, belonging to the Bulinae subfamily, whereas all the others are classified in the Planorbinae subfamily. Moreover, the bacterial microbiota of this species seems to be very specific, as suggested by the high abundance of *Mycoplasma*. Interestingly, individuals of the strain Bg BS90 also displayed strong dissimilarities with the other species and even the other strains of *B. glabrata*, with most of the dissimilarity values also ranging from 0.8 to 0.99.

As the different strains were maintained in separate tanks, we performed a common garden experiment to circumvent potential biases due to mollusk maintenance and tested whether the same microbial environment would lead to a homogeneous distribution of the bacterial communities between snail strains. This result confirmed a specificity of the microbiota by strain/species, suggesting that the importance of the host effect in microbiota composition is higher than the effect of rearing conditions. The microbiota can nevertheless vary during the host lifespan, with an initial recruitment of bacterial communities occurring during early development. It would be interesting to test the possibility of microbiota transfers from the environment in different developmental stages when the definitive flora is not yet fully established. A recent study showed a loss of microbial communities from one generation to the next in laboratory reared mosquitoes (Akorli et al., 2019), which presents another avenue for our model to be further investigated.

In both individual and common garden experiments, almost every individual of each strain grouped together in the dendrogram, supporting the specificity according to the host. In addition, the topology of microbiota dissimilarities was congruent with the mollusk phylogeny, despite a limited number of strains but that covers species, genera, and subfamilies of Planorbidae. This suggests a pattern of phylosymbiosis at the host species level among snail intermediate hosts of schistosomiasis. This has already been described in other models, for both vertebrates and invertebrates. In vertebrates, for example, a loose phylosymbiosis pattern was identified between 44 species of coral reef fishes and their skin microbiota (Chiarello et al., 2018), possibly related to a plasticity in the immune system. Host immune genes and other factors like nutrient production by the host and vertical transmission have also been hypothesized to explain phylosymbiosis between several populations of American pika, *Ochotona princeps* (Kohl et al., 2017). For invertebrates, this pattern was shown in three *Nasonia* species, in a controlled environment, with such a codiversification and coevolution that there is a lethality of hybrids from a breed between two *Nasonia* species (Brucker and Bordenstein, 2013). This codiversification, as a mechanism leading to phylosymbiosis, has also been hypothesized in a study comparing microbiota composition of 15 *Cephalotes* species (Sanders et al., 2014), whereas it would not be the main driver of this phenomenon in corals, in which phylosymbiosis would be led by other mechanisms like biogeography or host traits (Pollock et al., 2018). Similar findings of phylosymbiosis driven by the host have been identified between two different species of *Hydra* (Fraune and Bosch, 2007), and many studies have shown that the host genetic background shape the microbiota in numerous models (Chaston et al., 2016; Coon et al., 2016; Parker et al., 2017; Sánchez-Cañizares et al., 2017; Paniagua Voirol et al., 2018). In our model, this correlation between host and microbiota indicates that host phylogeny highly constrains the microbiota composition and structuration (Brooks et al., 2016; Chiarello et al., 2018). However, this pattern may not be ubiquitous, and a few studies on *Drosophila* (Chandler et al., 2011), mosquitoes (Osei-Poku et al., 2012), or flea beetles (Kelley and Dobler, 2011) identified no correlation between host phylogeny and microbiota composition. Nevertheless, we could not assess the phylosymbiosis pattern at a lower phylogenetic level (i.e., the strain), as we cannot determine the genetic distance between the different *B. glabrata* strains because of inbreeding in the laboratory and high differentiation between strains.

In our case, the phylosymbiosis pattern could not be considered as a hallmark of coevolution because we focused on the whole bacterial community of a host, with very complex interactions, and not on a specific symbiont. Here, we defined coevolution according to O’Brien et al. (2019), as a “*reciprocal evolution of […] a broad range of interactions such as predator–prey, host–symbiont and host–parasite interactions, or interactions among the members of a community of organisms such as a host and its associated microbiome*.” O’Brien et al. (2019) noticed that hosts and their symbiont phylogenies are often mirrored, which can be interpreted as a parallel divergence called a codivergence. This codivergence has often led to obligatory symbiosis, as is the case between pea aphids and bacteria from the genus *Buchnera* (Baumann et al., 2006) and is notably found in mutualistic symbiosis (O’Brien et al., 2019). In this case, the protagonists have a very close interaction, with participation in each other’s physiological mechanisms.

The host–microbiota specificity illustrates the high interaction between the snails and their bacterial communities, suggesting an impact of the latter on its host fitness toward several functions like nutrition, development, reproduction, and immunity. Given that, in this model, Planorbidae snails are intermediate hosts of *Schistosoma* parasites, it would be interesting to study the tripartite interaction between the trematodes, the mollusks, and their microbiota.

Indeed, previous studies highlighted a variation in the compatibility phenotype between different combinations of *B. glabrata* strains and *S. mansoni* parasites (Theron et al., 2014; Galinier et al., 2017). Moreover, *P. metidjensis* and *B. truncatus* are not compatible with the same *Schistosoma* species. This compatibility polymorphism can be seen as a hallmark of differences in immune capacities. As the phylosymbiosis pattern suggests a strong link between host and microbiota, the hypothesis of a relationship between the snails’ immune capacities and the composition of their microbiota can be made.

The protective role of whole microbiota (or gut microbiota) has indeed been shown in numerous models like the mosquitoes against dengue virus (Ramirez et al., 2012) or the honey bees with the augmentation in antimicrobial peptide production (Li et al., 2017). Another example is the microbiota of *Dysdercus fasciatus* that acts as a physical barrier to prevent the entry or attachment of a parasite (Onchuru et al., 2018). Chiu et al. (2017) also reviewed several examples of microbiota actions against pathogens, such as slowing or preventing the entry, installation, development, and expansion of pathogens. In some models, the microbiota has a direct effect against their hosts’ pathogens, producing effectors like reactive oxygen species (Cirimotich et al., 2011), or an indirect effect, promoting some immune pathway. Concerning the interaction between Planorbidae and *Schistosoma*, the immune mechanisms have been well studied; however, there are very few information concerning the tripartite interactions. Some immune genes located in a Guadeloupe resistance to parasite complex region have been shown previously to contribute in shaping microbiota (Allan et al., 2018), highlighting a link between microbiota composition and host immunity.

The present study highlighted a strong host–microbiota specificity, which confirms the link between host genetics, immune capacity, and microbiota composition. However, more information are needed to understand if there is a direct or indirect impact of microbiota on the host–parasite interaction.

The interaction between microorganisms and the host immune system can be complex. The microbiota stability can be affected upon parasite primo-infestation and challenge, suggesting a tight control of immune system on bacterial composition (Portet et al., 2018). The next step will be to compare the microbiota dynamics during an infection kinetic with several host/parasite combinations with different immunobiological interactions. Although a shift in microbiota composition during an infection associated with changes in snail immune gene expression was clearly established according to the host/parasite combination (sympatric/allopatric) (Portet et al., 2018), further studies are needed to clarify the link between microbiota and snail host immunity. Phylosymbiosis pattern is a hallmark of tight interactions between host and microbiota, suggesting the role of microbial communities on different host physiological functions, including immunity. This study thus paves the way for future studies to decipher the role of microbiota in host fitness, including the development and transmission of parasites.

## Data Availability Statement

The datasets generated for this study can be found in the Sequence read Archive repository under BioProject PRJNA 554540 (sequence data to be released upon publication).

## Author Contributions

CH, BG, RG, DD, and ET involved in the study concept and design. CH involved in sampling and data acquisition. CH, CC, and ET performed the data analysis. CH and ET drafted the manuscript. All authors contributed to the critical revisions and approved the final manuscript.

## Conflict of Interest

The authors declare that the research was conducted in the absence of any commercial or financial relationships that could be construed as a potential conflict of interest.

## Footnotes

1 http://bioinfo.genotoul.fr

2 http://www.phylogeny.fr

## Supplementary Material

FIGURE S1 Principal Coordinate Analysis (PCoA) of every individual from each strain in the common garden experiment. Each dot is an individual and each color a strain. The labels are displayed at the barycenter.

TABLE S1 OTU table with taxonomic affiliation and read numbers for each sample.

TABLE S2 Core microbiota by strain/species at the family level (representing at least 0.6% of the total sequence read number by strain). The families highlighted in blue are common to all the strains.

TABLE S3 OTUs significantly associated with each single host species using indicspecies.
