# Supplementary figures and images for: Schistosomiasis Vector Snails and Their Microbiota Display a Phylosymbiosis Pattern
Source: Front Microbiol. 2020 Jan 31;10:3092. doi: 10.3389/fmicb.2019.03092 (PMC7006369; doi:10.3389/fmicb.2019.03092)

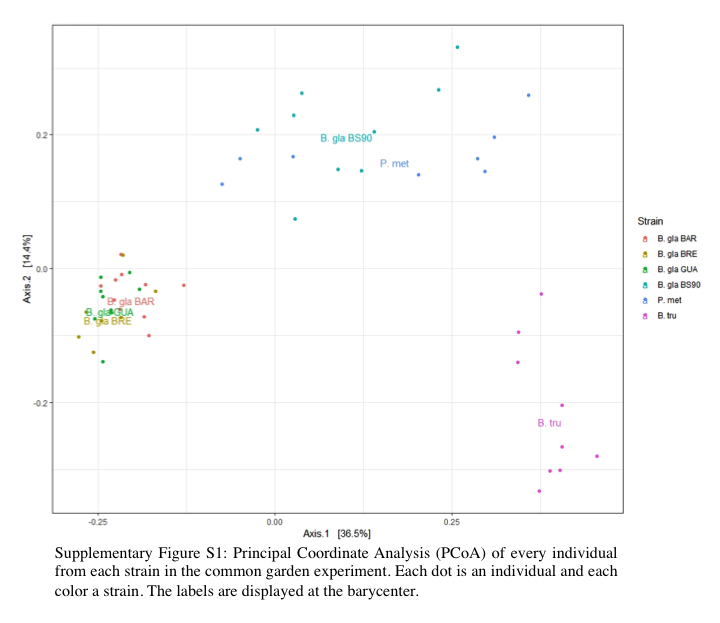

Supplement: FIGURE S1 — Principal Coordinate Analysis (PCoA) of every individual from each strain in the common garden experiment. Each dot is an individual and each color a strain. The labels are displayed at the barycenter. [file Image_1.TIF]
